# Bowel anastomosis leakage following endometriosis surgery: an evidence based analysis of risk factors and prevention techniques

**Published:** 2020-10-08

**Authors:** A Vigueras Smith, R Sumak, R Cabrera, W Kondo, H Ferreira

**Affiliations:** Department of Minimally Invasive Surgery Unit of Centro Hospitalar Universitário do Porto, Porto. Portugal; Department of Gynaecology and Minimally Invasive Unit, Vita Batel Hospital. Curitiba. Brazil

**Keywords:** Anastomotic leakage, bowel endometriosis, colorectal anastomosis, endometriosis

## Abstract

**Background:**

Deep endometriosis most commonly involves the rectosigmoid junction and its management often requires a colorectal resection. Anastomotic leakage is a severe complication after resection and affects 1-6% of the cases.

**Objective:**

To evaluate the risk factors related to anastomotic leakage following endometriosis sur-gery, its prevention techniques and the role of protective stomas.

**Methods:**

A comprehensive literature review was carried out for English-language publications in Pubmed and Google Scholar. We included all studies including the following MeSH terms and key words: Anastomotic leakage AND bowel surgery OR Endometriosis OR Colorectal surgery OR Bowel endometriosis. Two authors independently made a selection and analysed relevant abstracts according to the aim of this review.

**Results:**

Risk factors and preventive measures were categorised considering the patient condition, the intra- operative setting and the surgical procedure itself. Level I and II recommendations include modifiable risk factors such as the use of stapled or handsewn anastomosis; intra-operative air leak test to check the integrity of the anastomosis; systematic use of pelvic and trans-anal drainage; application of protective or ghost ileostomy in low rectal resections; vaginal closure before the bowel resection; use of oral antibiotics the day before surgery and performing partial mesorectal resection near the bowel wall. Diverting stomas may decrease the morbidity and the clinical consequences of leakage over 65% of low rectal resections but may cause significant adverse effects.

**Conclusion:**

Evidence-based protective actions are crucial to reduce clinical consequences of anastomotic leakage and to minimise the use of protective stomas in endometriosis surgery.

## Introduction

Bowel endometriosis is defined as the presence of endometrial-like glands and stroma infiltrating the bowel wall and affects 5% to 12% of patients with deep infiltrating endometriosis (DIE) (Abo et al., 2018). When surgery is indicated, amongst other challenges, anastomotic leakage (AL) appears as a major life-threatening complication affecting around 1-2% of segmental resections, significantly increasing morbidity, mortality and reoperation rate (Richards et al., 2012). Due to the high number of bowel endometriosis cases requiring surgery in current practice, it is necessary to have a thorough knowledge of AL presentation and its preventive methods in order to reduce their numbers to a minimum. The primary objective of this review is to analyse the currently available information relating to AL risk factors and preventive techniques following surgical treatment of bowel endometriosis, and the role of systematic use of protective stomas.

## Materials and methods

A comprehensive review of the literature was carried out for English publications in Pubmed and Google Scholar relating to bowel anastomotic leakage following endometriosis surgery. We included all studies found under the search of following MeSH and the keywords terms: Anastomotic leakage AND Bowel surgery OR Endometriosis OR Colorectal surgery OR Bowel endometriosis.

Initially, a structured investigation question was created using the PICO strategy as shown in Table I.

**Table I:** Structured investigation strategy used in this review.

P.I.C.O. Structured Investigation Question		
P	(Patient/Problem)	Women who underwent colorectal surgery for endometriosis and suffering anastomotic leakage
I	(Patient/Problem)	Shaving, discoidal and/or segmental bowel resection for symptomatic endometriosis
C	(Comparison)	Oncologic colorectal resection and anastomosis
O	(Outcome)	Identification of risk factors and determining effectiveness of preventive techniques

AL is defined as the leakage of luminal contents from the surgical join between two hollow viscera (Peel and Taylor 1991). The luminal contents may emerge either through the wound or at the drain site, or they may collect near the anastomosis causing fever, abscess, septicaemia, metabolic disturbance and/or multiple organ failure. The asymptomatic leak of luminal content from the anastomotic site into an adjacent localised area detected by imaging exams should be recorded as a sub-clinical leak (Bruce et al., 2001).

Diagnosis frequently encompasses clinical, biochemical and imaging exams. Leakage may present itself as pain, fever or feculent discharge from a drain. Clinical signs may include tachycardia, abdominal tenderness and signs of peritonitis (Table II). Laboratory findings usually show an elevated white cell count and an increase in acute-phase biochemical markers (pro-calcitonin, C-reactive protein) during the first 3 to 5 days postoperatively. Radiological investigations (CT scan, water-soluble contrast media enema, contrast media MRI) show signs of a fluid collection which may contain gas or contrast in proximity to the anastomosis (Kanellos et al., 2004).

**Table II:** Clinical symptoms and radiologic signs of anastomotic leak.

Area	Findings		
Clinical Exam	Abdominal pain	Fever	Altered mental state
	Tachycardia - Tachypnoea	Peritonitis findings	Feculent drainage
	Rectal pus/blood discharge	Wound pus/faecal discharge	Abdominal mass (Abscess)
Imaging	Loculated fluid collection	Gas containing collection	Contrast collection

Bruce et al. (2001) proposed three types of lower gastrointestinal AL based on signs, symptoms and severity, independent of the level of the colorectal anastomosis.

1. **Radiological:** No clinical signs.
2. **Clinical-Minor:** Leakage of luminal contents through the drain/wound - local inflammation - fever (>38°C) - tachycardia - leukocytosis (over 10.000/ litre) - faecal purulent discharge from drain/wound (abscess).
3. **Clinical-Major:** Same as minor plus severe disruption of the anastomosis.

There are known risk factors for leakage inherent to the patient condition, intra-operative setting and surgical technique (Phillips 2016). A summary of these are presented in Table III (Law et al., 2000; Lipska et al., 2006).

**Table III:** Risk factors for bowel anastomotic leakage.

Setting	Risk Factors		
Patient Condition	Gender - male	Age > 60	Radiotherapy
	Malnutrition/Weight loss	Smoking habit	Steroid use
	Renal failure	Diabetes mellitus	Cardiovascular disease
	Alcoholism	Concurrent bowel disease (Crohn disease, diverticulitis)	Anaemia
	Chemotherapy	Ascites	Cardiovascular disease
Peri-operative Setting	Prolonged surgical time	Restriction or overload of intravenous fluids	Use of pressor agents
	High blood loss and transfusions	Emergency Surgery	Asa classification > 2
	Multifilament absorbable threads	Butressing anastomosis	Left colon anastomosis
Surgical Technique	Low or ultra-low anastomosis	Double-layer bowel closure	Nodule size over 3 cm diameter
	Concomitant opening of the vagina (RVF)	Mechanical bowel preparation	Segmental bowel resection
	Positive air-leak test	Total mesorectal excision	

A thorough analysis of the principal risk factors will be presented later (See Prevention of Risk Factors).

## a. Anastomosis leak in bowel endometriosis surgery

### Scope of the problem

Bowel endometriosis, the most common extragenital endometriosis location, is defined as endometriotic infil-tration of the intestinal muscle layer and affects between 5% to 12% of patients with DIE (Abo et al. 2018;; Nezhat et al. 2018). It is usually a multi-focal and multi-centric disease involving predominantly the rec-tosigmoid junction and the rectum (70%- 90%), infiltrating progressively from the serosa toward bowel lumen, which is rarely affected by the nodules (Nezhat et al., 2011; Bertocchi et al., 2019). Preoperative diagnosis is based mainly on the presence of specific symptoms (cyclical functional bowel disorder, rectorrhagia, dyschesia, catamenial diarrhoea, constipation, blood in the stool, bloating), signs (palpable nodule or thickened area along with the utero-sacral ligaments, uterus, vagina or recto-vaginal septum on recto-vaginal examination) and imaging tests, such as pelvic magnetic resonance imaging (MRI), transvaginal and transrectal ultrasound (US). Imaging should be able to specify the tumour size, number, location, depth of infiltration and degree of luminal stenosis to choose the best surgical option (Nisenblat et al., 2016; Nezhat et al., 2018). Transvaginal US has been shown to be highly specific (92-100%) and sensitive (71-98%) in obtaining these tumour variables, according to published systematic reviews (Hudelist et al., 2011).

Surgery is indicated in symptomatic cases or when an intestinal obstruction is present. The therapeutic options include nodulectomy (shaving, mucosal skinning, discoidal resection) and segmental resection. Shaving is defined as the superficial serosal and subserosal bowel excision, not requiring suturing or closure. The mucosal skinning (also known as “rectal partial thickness excision” or “deep rectal shaving”) includes excision of the rectal muscularis without opening the mucosa, requiring suturing. Finally, disc excision encompasses a full-thickness resection of the entire anterior rectal wall, while segmental resection includes removal of a whole bowel segment (Darai et al., 2017).

The correct treatment choice is selected after an assessment of the disease variables such as nodule diameter, number, location, depth of infiltration, and presence/absence of luminal stenosis (Nezhat et al., 2018). Segmental resections have been performed since the early 1900s. They are mainly indicated in cases where implants are more significant than 3 cm in diameter, have submucosal and/or mucosal involvement, cause stenosis of more than 40% of bowel lumen and are multifocal or located in the sigmoid colon (or higher), as well as following persistent symptoms after nodulectomies (Nezhat et al., 2018; Hudelist et al., 2018). Patients undergoing this procedure are at increased risk of postoperative morbidity, including permanent stoma (and its related complications) and anastomosis line problems (Nezhat et al., 2018). AL is one of the most serious complications of the latter, reaching 3% to 6% in the segmental resections (Abo et al., 2018), but according to some authors can reach up to 20% depending on the definitions used, level of anastomosis and length of the follow-up (FU) (Umanskiy and Hyman, 2019). Its occurrence determines an immediate increase of mortality (reaching up to 15%), severe morbidity, elevated re-admissions and re-operations, more extended hospital stay, higher cancer recurrence (in the oncologic setting) and lower quality of life (Petersen et al., 1998; Richards et al., 2012).

### Evidence of anastomotic leakage after bowel endometriosis treatment

#### 1. The importance of operative standard disease classification

Deep endometriosis is prevalent and commonly a multi-organ disease. With these characteristics, the lack of a universal standard operative method to report it becomes a significant disadvantage. Standardisation of a procedure helps to eliminate errors due to omission or admission, provides benchmarks to determine when corrective actions are required, facilitates training by providing regular steps that can be taught, practised and evaluated, creates a common language to describe a specific process that can be understood and commu-nicated between surgical teams and preserves the knowledge in time. Therefore, maximal effort must be put in to correctly classify DIE as a very first step in each surgical case.

The most commonly used system, the American Society of Reproductive Medicine (revised) classification, has the drawback of not considering deep endometriosis and does not correlate the severity of clinical symptoms (pain) with the extent of the disease (Vercellini et al., 2007). Recently, the recommendations of the Working Group of the ESGE, ESHRE and WES (2020) have focused on the advantages of other classification systems, especially the ENZIAN score (Tuttlies et al., 2005), since recent evidence has shown that this classification significantly correlates the extent of the disease, difficulty and length of the surgery and symptoms. The ENZIAN score classifies clinical endometriosis findings considering two factors: the three-dimension anatomical localisation (3 compartments plus uterine and another extra genital DIE) and implant size (<1 cm, 1-3cm, > 3 cm). Finally, it allows the scoring of each compartment into three grades of severity (I-II-III), providing an ex-cellent morphological description and an adequate correlation between clinical symptoms and severity of involved compartments (Working Group of ESGE/ESHRE/WES, 2020).

#### 2. Laparoscopy or laparotomy?

After the first laparoscopic bowel resection for endometriosis was performed in 1988, several well designed prospective studies have demonstrated the advantages of laparoscopy versus laparotomy in the treatment of bowel DIE, including less blood loss and hospital stay, post-operative complications and higher pregnancy rate, without significant differences in the long term post-operative symptoms control (Nezhat et al., 2018; Nezhat et al., 2011). Thus, laparoscopy is the most preferable approach for this pathology.

#### 3. Shaving, discoidal or segmental resection?

Anastomotic leakage is a complication that can occur after any of these bowel procedures. Even when segmen-tal resections tend to show a higher absolute risk of leakage than nodulectomies in retrospective data, this is not necessarily true and must be carefully interpreted. So far, the only randomised controlled trial (RCT) available comparing functional outcomes after conservative (shaving, disc excision) or radical (segmental resection) bowel DIE treatment did not find significant differences in “complications related to stoma repair” (leakage, haemorrhage, hernia) between the groups (7.4% vs 3%) (Roman et al., 2018).

Segmental resection implies complete resection of the affected bowel segment with subsequent primary end-to-end, end-to-side or side-to-side anastomosis, usually requiring extensive dissection of para-rectal spaces where important vascular and nerve structures are located. If they are damaged, severe morbidity including bowel ischemia, fistulas and anastomotic leakage can develop (Nezhat et al., 2018). Indications for this procedure were previously mentioned and must consider the number, size and depth of the nodules, associated fibrosis, rectal circumference involvement and distance from anal verge (Abrao et al., 2015).

Abo et al. (2018) compared the post-operative outcomes of the main surgical techniques for treating 364 bowel DIE cases (shaving, discoidal and segmental resection) and reported just one case of leakage (0.3%) in the segmental resection arm, requiring second surgery and stoma formation. Interestingly, 6.6% of all the cases presented with pelvic abscess without evidence of leakage or fistula. Similar results were found by Mohr et al. (2005), where among 187 DIE bowel cases (1000 shavings, 39 discoid and 48 segmental resections), just one case (0.53%) of leakage occurred, again in the segmental resection group (Mohr et al., 2005).

Donnez and Roman (2017) reviewed the peri- operative outcomes of different DIE bowel surgeries, including 61 studies and 10,848 patients. They showed that the rates of urinary retention (0–17.5%), ureteral lesions (0–2%), anastomotic leakage (0–4.8%), and pelvic abscesses (0–4.2%) were all higher with bowel resection than with the shaving technique or disc excision. There were no cases of leakage in the shaving (25 studies/6491cases) and the discoidal resection arms (10 studies/455 cases), while in the segmental resection (26 studies/ 3902 cases) the mean leakage rate was 1.72 %. However, the rate of rectovaginal fistulas in the disc excision group was threefold higher (3.6%) than the shaving procedures (1.3%) and almost equal to the rate seen in the segmental resections (3.9%). Careful interpretation of this data is necessary as it is mostly retrospective and leakage definition/inclusion criteria are heterogeneous among studies. Although this result is consistent with a recent narrative review by Nezhat el al. (2018), surgeons must understand that this complication can occur after any surgical treatment of bowel endometriosis.

#### 4. Main series of laparoscopic DIE bowel resection and anastomotic leakage

##### 

Up until now, 30 series with over 5500 cases of bowel resection have been published (Donnez and Roman, 2017; Bertocchi et al., 2019). All studies are retrospective, variable in the number of cases (6 to 774) and heterogeneous in reporting the intra- and post-operative complications. The pooled data show an overall leakage rate of 1.7 %, ranging between 0% and 4.8%. Since a major part of the data for this topic comes from descriptive and analytical-observational studies, retrospective in their temporality, here we analyse the largest ones briefly. The main retrospective series, based on the number of patients included in the analysis, were published by Bertocchi et al. (2019), Ruffo et al. (2010), Roman et al. (2017), Minelli et al. (2009), Malzoni et al. (2016) and Keckstein and Wiesinger (2005). Moreover, data coming from 2 meta-analyses (De Cicco et al., 2011; Meuleman et al., 2012) complement our knowledge about this complication. For this review, the internal validity analysis of each article was not performed. Therefore, we encourage clinicians to read the studies indepth when necessary.

##### Retrospective studies

Bertocchi et al. (2019) published the largest series with 1643 segmental resection for bowel DIE. By using the Negrar method (segmental resection without ligature of inferior mesenteric artery), this group focused on the evaluation of the rate of anastomotic stenosis. They found 6.3% had symptomatic anastomotic stenosis, of which 1.9% presented with AL. They identified that the presence of a protective ileostomy was the only significant modifiable risk factor related to anastomotic stenosis, present in 32% of stenotic cases.

Ruffo et al. (2012) presented 750 laparoscopic mid/low rectal (segmental) resection and transanal “end-to-end” anastomosis cases. They reported 3% as having AL and 2% as having recto-vaginal fistula (RVF). An abdominal drain was maintained for a median of 4.5 days (1-15) and temporary ileostomy was performed in 14.5% of the cases. All cases of leakage occurred in non-ileostomized patients. This data was complemented and presented by the same group in 2014, this time with a total of 900 cases. However, no rates of leakage or RVF were noted (Ruffo et al., 2014).

Similarly, in 357 bowel resections and end-to-end anastomoses (89.6% by using endo-anal circular stapler and 10.4% manual hand-sewn via mini-laparotomy), Minelli et al. (2009) reported 1.1% as having leakage and 3.9% as having RVF. The anastomoses were predominantly low (83.5%) and ultra-low (7.6%). Temporary stoma was performed in 11.5% of cases while vaginal opening was necessary for 31%.

Roman et al. (2017) evaluated the post-operative surgical outcomes of 1135 cases of bowel DIE treated by three approaches; laparoscopy (82.2%), robotic- assisted (9.7%) and laparotomy (8.1%). Treatments in-cluded shaving (48.1%), segmental (46.8%) and discoidal resection (7.3%). Anastomotic leakage presented in 0.8% of segmental resections, while pelvic abscess occurred in 3.4%.

Malzoni et al. (2016) analysed the post-operative complications of 248 segmental bowel resections. Anastomotic leakage was present in 1.6% of the cases, most frequently between days 3 and 5. Moreover, peritonitis without laparoscopic signs of leakage was observed in 0.8% of the cases, and resolved by using antibiotics and protective ileostomy for four months. RVF developed in 2.4% of the cases, including all cases with ultra-low anastomosis, concomitant vaginal resection and without a temporary stoma. The study concluded that in cases of ultra-low rectal resections, termino-lateral anastomosis and temporary protective stoma must be highly considered to avoid these complications.

Similarly, Keckstein and Wiesinger (2005) analysed 202 bowel resections and reported 3% of cases as hav- ing leakage, and 1% as having para-rectal abscesses. Apart from pre-operative antibiotics, bowel prepara- tion and an air-leak test, no other protective procedures were done. The authors consider 3% of AL as low risk, concluding that laparoscopic segmental resection is effective and secure, with significant benefits regarding the patient’s quality of life.

Finally, Dousset et al. (2010) analysed 100 cases of bowel resections with anastomosis and reported 2% as having AL and 4 % as having RVF. Omental flap interposition and pelvic drainage were done in 100% of cases, while protective stoma was carried out in 96 patients. All these patients had total mesorectal excision including inferior mesenteric artery ligation, and the mean anastomosis distance from the anal verge was 3.6 cm (all of them less than 6 cm), two crucial factors related to leak risk.

##### Systematic reviews

The systematic review of De Cicco et al. (2011) includes 1889 segmental resections for bowel endometriosis and reports 2.7% as having leakage and 1.8% as having fistulas, directly related with the level of the anastomosis; the lower the anastomosis, the higher the risk of postoperative leakage. They did not report the use of any specific protective procedures.

Similarly, Meuleman et al. (2011) reviewed the surgical outcomes of bowel DIE surgical treatment, including over 2770 patients. The rate of leakage was 1.5%, with 2.7% having RVF, and 0.34% with an abdominal abscess. No information about leakage/fistula preventive measures was published. Nevertheless, the authors recommend the use of systematic protective ileostomy in cases of concomitant vaginal and rectal resections to reduce the risk of fistulas and pelvic abscess.

## Diverting bowel stoma in colorectal surgery

For low and ultra-low colorectal anastomosis at high risk of developing fistula and leakage, the use of temporary protective ileostomy is usually recommended in order to prevent these complications. However, it is asso-ciated with stoma-related risks, such as hernia, retraction, stenosis, sinus formation, dehydration, prolapse and necrosis (Bertocchi et al., 2019). Randomised controlled trials (RCTs) and meta-analyses have demonstrated that the use of defunctioning stoma in low colorectal anastomosis may reduce the morbidity and clinical consequences of leakage (up to 68% reduction of clinically symptomatic AL and 73% fewer re-operations). However, they do not seem to reduce the likelihood of occurrence itself or the mortality rates (Huser et al., 2008; Matthiessen et al., 2007; Boyce et al., 2017)

Considering that endometriosis is a benign disease in young and healthy people, adequate case selection and patient counselling is crucial (Minelli et al., 2009). Treatment must always be tailored according to the patient’s disease, desires and expectations.

## a. Stoma complications

The stoma can significantly affect patients’ quality of life and sense of well-being, also burdening the health care system (Krishnamurty et al., 2017). Complication rates range from 20% to 70% and can be grouped into early (less than 30 days from surgery) and late-onset (after 30 days) (Shabbir and Britton, 2010). Early complications include retraction, ischemia, necrosis, para-stomal abscess and mucocutaneous separation, while late ones are mainly prolapse, varices, para-stomal hernia and retractions.

## b. When to perform a temporary bowel diversion

### 

Whether to perform a bowel stoma or not depends on the surgical team preferences, but must be guided by some specific factors:

### 1. Level of anastomosis

The site and height of anastomosis are crucial. It is well accepted that the serosal layer has a critical role in anastomotic healing. Since the lower rectum is lacking in this layer, a higher risk of leakage is expected at this level (Moran et al., 2017). Furthermore, lower lesions usually require extensive para-rectal dissection which can harm vascular structures, compromising the final vascularisation of the bowel at the anastomotic line, increasing the risk of leakage and temporary defunctioning stoma (Nezhat et al., 2018).

**Figure 1:**
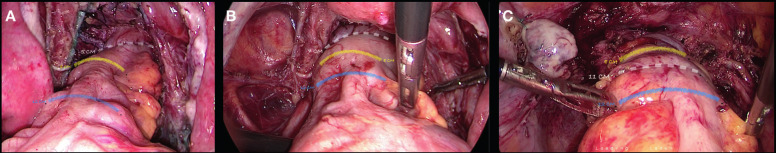
Bowel anastomotic levels.Three consecutive images representing the three levels of anastomotic lines according to their distance from the anal verge. A: Medium or High anastomosis (> 8 cm from AV) ; B: Low anastomosis (5 to 8 cm from AV) ; C: MUltra-Low anastomosis (< 5 cm from AV). White line: Anastomotic line. Yellow line: 8 cm form AV. Blue line: 12 cm from AV.

The level of the anastomosis can be classified into three types according to their distance from the anal verge (AV) (Figure 1):

- **High/Medium:** Equal or more than 8 cm.
- **Low:** Less than 8 cm but more than 5 cm.
- **Ultra-low:** 5 cm or lower.

Even though initial studies did not show a significant relationship between the rate of fistulas and level of anastomosis, probably due to the bias of using protective ileostomy in low and ultra-low anastomosis (Mereu et al., 2007), recent evidence has consistently shown that the rate is significantly higher in the left side of the colon, and specifically in those performed within 10 cm from AV (Bakker et al., 2014; Trencheva et al., 2013; Abrao et al., 2015). Furthermore, several prospective studies have shown that the lower the anastomosis; the higher the risk of leakage (Park et al., 2013). The leakage rate is up to 3.4 times higher for tumours located less than 7 cm from the AV (Hamabe et al., 2018) and ten times higher for those located under 5 cm of the AV (Choi et al., 2010).

### 2. Total or partial meso-rectal resection (oncologic versus benign pathology)

**Figure 2:**
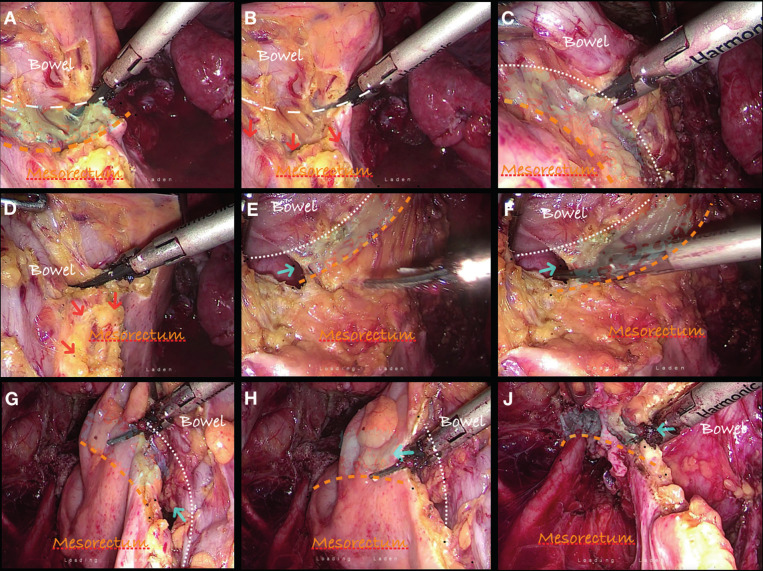
Meso-rectal resection for bowel DIE resection. 9 consecutive images showing the technique for the meso-rectal dissection in the endometriotic scenario. Since this is a benign disease, dissection must be performed as close as possible to the bowel in order to obtain maximum preservation of irrigation and innervation in between the white and the orange lines. Dissection must be performed up to 2 cm away from the DIE nodule edges.

Total meso-rectal excision (TME) is the standard treatment for locally advanced rectal cancer, reducing the risk of recurrence and improving global prognosis (Bianchi et al., 2014), while benign conditions need just partial meso-rectal excisions near the bowel. When rates of leakage in bowel endometriosis surgery are com-pared to rectal cancer, percentages are consistently higher in the latter, reaching up to 17% (Sartori et al., 2011). A meta-analysis reports a leakage rate of post-TME ranging from 5.4% to 5.8% (Hua et al., 2014). This is explained in part by the fact that endometriosis is a benign disease affecting healthy young women without major comorbidities. Additionally, and although surgical techniques for segmental resection vary widely among different teams, DIE bowel resection could encompass a “nerve-vessel sparing segmental resection”, where mesorectum resection is limited to the macroscopic DIE infiltration area and cutting of the inferior mesenteric vessels is avoided. This strategy results in a tubular fashion dissection which spares all the fatty tissue, hypogastric nerve plexus and vessels lateral to the bowel segment resected. Despite the absence of solid evidence about the benefits of this type of segmental resection compared to others, the theoretical im-provement of the anastomosis vascularisation could enhance the bowel healing process and reduce the risk of leak and micro-leaks (Hudelist et al., 2018) (Figure 2).

### 3. Result of the intra-operative air-leak test

**Figure 3:**
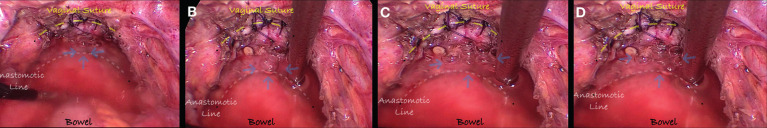
Positive air-leak test. 4 consecutive images demonstrating a positive air-leak test. After bowel occlusion with laparoscopic atraumatic grasper distal to the bowel anastomosis, 60-400 cc of air is directly inserted trans-anally to distend the rectum. The blue arrows show the bubbles coming from the mechanical anastomosis dysfunction.

Currently, an air leak test is one of the most frequent intra-operative tests for evaluation of mechanical anastomosis competence, as well as diagnosis and treatment of occult disruptions (Umanskiy and Hyman, 2019). A positive test leads to the performing of further procedures, such as reinforcement stitches or protective ileostomy to avoid anastomotic complications (Nachiappan et al., 2014) . Even though it is widely used, a unique and clear definition and standardisation of the test does not exist. Many different techniques in terms of in- sufflation methods (syringe, catheters, endoscope, sigmoidoscope, rectoscope, proctoscope), solutions (air, saline solution) and volumes (60 mL to 400 mL) have been described (Wu et al., 2016). However, clinicians must consider that colorectal anastomosis support can stand pressures around 70 to 184 mmHg; therefore, a volume near 400ml must be injected carefully under barometric intra-luminal measurement to avoid damage of anastomosis (Schwab et al., 2002). A recent meta-analysis of 20 studies with 5283 patients evaluate their role in the prevention of AL. Although the studies are biased, the rate of leakage after performing the air- leak test is consistently lower than those without testing (OR: 0.61), but not statistically significant (p=0.15). Nevertheless, among all the patients with the air-leak test performed, those with a positive test have significantly higher chances of presenting leakage than those with a negative one (4.2% vs 11.4%) (Wu et al. 2016). Surgeons must be aware that this test detects only mechanical disruption of the anastomosis, leaving out other pathologic mechanisms of AL such as healing disturbances or infection. In conclusion, the systematic use of this test does not significantly reduce the rate of leakage. However, its use is highly recommended since, in case of a positive result, the risk of leakage rises dramatically, and further protective procedures must be considered (thorough revision of anastomosis, suture reinforcement, redoing anastomosis, oversewing and re-testing) (Figure 3).

### 4. Deep endometriosis nodule size

Studies in colorectal cancer suggest that the nodule diameter may be a predicting factor for AL. With the in-crease in lesion size, intra-pelvic manipulation becomes restricted and rectal transection is more challenging, starting from 3 to 4 cm diameter (Eberl et al., 2008; Zhu 2010). Thus, when tumours are bigger than 5 cm, a 4-fold higher risk of leakage is seen (Kawada et al., 2014). In endometriosis, the nodule size is closely related to the type of surgical bowel treatment, and therefore, the expected risk of leakage. According to Abo et al., (2018) shaving techniques are usually performed in nodules up to 3 cm. In comparison, discoidal or segmen-tal resections are conducted on nodules more prominent than 3 cm (Ferrero et al., 2009; Ghezzi et al., 2008). Since segmental resection appears to have higher rates of AL than nodulectomies, the bowel DIE nodule size seems to be an indirect risk factor of AL when over 3 cm.

### 5. Type of bowel surgery: Shaving, Discoid or Segmental Resection

As previously noted, AL events can occur in any of these surgical modalities, but predominantly in the low/ultra-low segmental bowel resection (See anastomotic leak in bowel endometriosis treatment).

### 6. Concomitant vaginal resection

**Figure 4:**
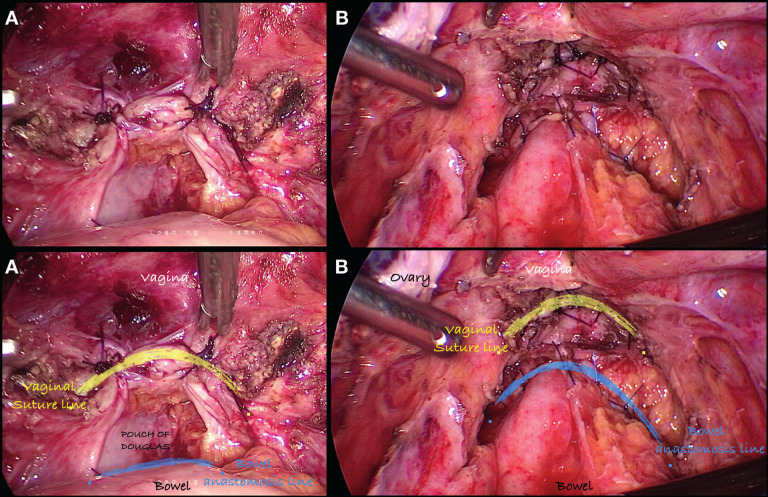
Concomitant bowel and vaginal resection . Images showing the anatomical relationship between vaginal closure and bowel anastomotic line. Upper set without annotation, lower set with annotation. Closer location increases the risk of rectovaginal fistula. A:Vaginal cuff closure after total laparoscopic hysterectomy and bowel anastomosis ; B: Vaginal closure after resection of vaginal DIE nodule plus bowel anastomosis.

Concerning RVF, concomitant vaginal resection appears to be a predisposing factor. According to Abo et al (2018) RVF was present in 3.8% of 364 bowel resections (without any differences between the type of bowel resection) with 50% of the cases having concomitant vaginal resection. It is always recommended to perform vaginal closure before bowel resection, and in cases of low bowel anastomosis, closing the vaginal opening and making interposition of the omental flap should usually be considered (Minelli et al., 2009; Ruffo et al., 2010) (Figure 4). However, for isolated anastomotic leakage, vaginal opening does not appear to be a primary factor.

### 7. Surgeon’s experience

Surgeon’s experience is still one of the significant factors in deciding whether to perform a stoma or not. Colo-rectal surgery is an advanced and difficult procedure which expose patients to a relatively high risk of severe complications. Thus, it should be performed in expert centres to reduce the instances of such complications . The French group of Bendifallah et al. (2017) analysed the relationship between case volume (rectum and sigmoid colon DIE) and incidence of complications, establishing an optimal cut-off value of 20 cases a year per centre and 7-13 procedures a year per surgeon for significant reduction of grade III and IV complication rates. It is clear that this type of colorectal surgery is certainly not an innocuous procedure and an evidence-based approach in the decision making should be adapted.

## Prevention of colorectal anastomosis leak

Up to now many techniques have been proposed to prevent or reduce the rate of leakage, but many of them do not have a corresponding evidence-based background (Table IV).

**Table IV:** Classical preventive techniques for anastomotic leakage.

Setting	Actions		
General	Smoking and alcohol cessation at least 4 weeks pre/ post-operative	Withdraw steroid use pre-operative	Schedule surgery at least 4 weeks after chemotherapy
	5-7 days of immune-modifying nutritional supplementation in malnutrition	Rationale use of NSAIDs	Systematic oral bowel preparation
Intra-operative	Short surgical time	Restricted blood transfusion	Normotension during surgery
	No tension, no overlap and adequate perfusion of anastomotic line	Avoid opening the vagina	Omentoplasty
	Single layer continuous closure	Monofilament delayed absorbable threads	Pelvic and transanal drainages
	Limited use of pressors	Re-enforce anastomosis when air leak test (+)	Diverting stoma

After evaluating the literature, we were able to classify the general and specific techniques for the prevention of leakage. Following the Canadian Task Force Levels of Evidence, we present the classification of these techniques based on the level of evidence (Burns et al., 2011).

## a. Control of general risk factors

### 

Modification of adjustable risk factors is important. Phillips (2016) gives simple recommendations for individual modifiable risk factors.adjustable risk factors is important. Phillips (2016) gives simple recommendations for individual modifiable risk factors.

### Alcohol

Since the intake of over 105g/week is associated with an increased risk of leakage, prompt discontinuation of alcohol intake is recommended in the pre- operative period (Sørensen et al. 1999).

### Malnutrition

Malnutrition impairs anastomotic healing by affecting collagen synthesis and fibroblast proliferation. Protein deficit (albumin < 3.5 g/L) and pre-operative weight loss are associated with low collagen levels and reduced bursting strength in the colonic anastomosis, leading to significantly higher rates of AL (Zhu 2010; Yadav et al., 2014; Turrentine et al., 2015; Mäkelä 2003).

Enteral supplementation significantly decreases the risk of surgical complications (including leakage) (Bozzetti et al., 2001), especially when immune-enhancing components, such as glutamine, arginine, n3-fatty acids and RNA are added Alcohol of peri-operative total parenteral nutrition is still inconclusive (Burden et al., 2012). In summary, pre- operative enteral “immune-enhanced” nutritional supplementation in malnourished patients is highly recommended (Cerantola et al., 2011).

### Smoking

Smoking increases the risk of leakage up to 4 times. Several studies have independently cited this as a factor, including both the active and previous smoking history (more than 40 pack-year) (Kim et al., 2011; Baucom et al. 2015). Since short term cessation does not reduce the risk, at least 4-8 weeks pre-operative suspension is recommended (Thomsen et al., 2014).

### Non-steroidal anti-inflammatory drugs (NSAIDs) use

Theoretically, NSAIDs increase the risk of leakage by generating a downregulation of prostaglandin expression and corresponding hydroxyproline levels, harming the normal healing process (İnan et al., 2006). Alt-hough initial studies were contradictory (Holte et al. 2009; Kverneng Hultberg et al., 2017), a systematic review and meta-analysis including eight studies and 4,568 bowel resections (99% of colorectal anastomosis), showed that overall use of NSAID was significantly associated with AL (OR:2.14), predominantly seen in non-selective NSAIDs. Nevertheless, considering the severe bias and heterogeneity of the studies, the results must be interpreted with caution. Careful prescription of NSAIDs to patients with pre-existing risk factors of leakage is advised (Bhangu et al., 2014).

### High Body Mass Index

Several studies report that BMI ≥ 35 kg/m2 is independently associated with AL (Silva-Velazco et al. 2016), significantly higher in the obese group (> 30 kg/m2 ) than in the non-obese (< 24.9 kg/ m2 ) and overweight (25 to 29.9 kg/m2 ) groups (Akiyoshi et al. 2011).

### No Bowel preparation

Pre-operative workup of colorectal surgery usually includes mechanical and enteral bowel preparation. In the last decade, there have been several studies which show no benefits of mechanical procedures in the prevention of leakage. Furthermore, this procedure carries risks of electrolyte disturbances and clostridium difficile infection. The classic French GRECCAR III RCT compared bowel preparation versus no preparation in 178 rectal cancer surgeries, and demonstrated that mechanical methods reduce the overall rate of septic complications, but not anastomotic leakage (Bretagnol et al., 2010). Hence, based on large RCTs and systematic reviews, avoiding this intervention is currently recommended (Slim et al., 2009; van’t Sant et al., 2015; Meyer et al., 2019).

In contrast, the policy of using non-absorbable oral antibiotics on the day prior to surgery is still highly rec-ommended since there is plenty of evidence of their benefits in reducing surgical site infections and AL in colonic surgery (Scarborough et al., 2015; Garfinkle et al., 2017). In a retrospective study that included 5291 patients, 62.5% of whom underwent colorectal surgery, oral antibiotics were associated with lower rates of surgical site infection and AL (Morris et al., 2015). Recent evidence coming from one meta-analysis and one retrospective analysis of over 8400 colorectal anastomoses confirm that this intervention significantly reduces the rate of leakage and surgical site infection (Morris et al., 2015).

## a. Control of general intra-operative factors

### Fluid restriction

Appropriate fluid administration should be part of intra-operative and post-operative care since there is evi-dence that both over-hydration and restriction are directly associated with a high risk of AL after colecto-my/gastrointestinal surgery (Schnuriger et al., 2011). The National Institute for Health and Care Excellence in 2011 recommended the use of goal-directed therapy to reduce post-operative complications. Nevertheless, there are no studies that prove it reduces AL rates (Futier et al., 2010).

### Hypotension

Patients with prolonged diastolic blood pressure drops have a 3-fold higher risk of AL (Post et al. 2012; Choudhuri et al., 2013). Similarly, patients who have post-operative treatment with vasopressors have a three to four-fold increase in AL rate, directly related to the time exposure to these drugs (Zakrison et al., 2007).

### High blood loss and transfusion

Higher intra-operative blood loss is associated with increased risk of AL (Defazio et al., 2014) by reducing colonic blood flow at the anastomotic level, leading to impaired wound healing and tissue necrosis (Irwin et al. 1990). Furthermore, blood transfusion in the perioperative period also increases the risk between 2 and 10-fold (Krarup et al., 2012). Moreover, the risk of blood-borne infections associated with transfusions is another reason for the use of a restrictive rather than liberal transfusion protocol (Burden et al., 2012).

### Anaemia

Inadequate perfusion and partial O 2 pressure are responsible for impaired wound healing. Normal pre- operative levels of haemoglobin must be checked and corrected when needed. A retrospective analysis of over 1200 major abdominal surgeries found that haemoglobin levels under 8g/dl independently increase the rate of AL 1.91 times (Choudhuri et al., 2013).

### Longer operative time

Duration of surgery is positively correlated with postoperative morbidity in both major and minor proce-dures (Scott 1982). The retrospective analysis of Silva-Velazco et al. (2016) found an AL increase of 3% for every 30 minutes of surgical time. The threshold for an increased chance of leakage was between 220 to 300 minutes (Huh et al., 2010).

### Poor intra-operative perfusion of anastomic line

It has been suggested that poor perfusion of anastomotic site as demonstrated by indocyanine green (ICG) may increase the risk of AL. Apart from the use of fluorescence-guided surgery for the detection of superficial endometriosis, intra- operative ICG assessment of the bowel wall (after shaving procedure) or the anastomotic line (after bowel DIE resection) vascularisation is a potential tool that might be helpful in confirming com- plete macroscopic resection of the disease and reducing the rate of bowel perforations. Therefore, the two theoretical benefits are choosing the adequate transecting line and evaluating the rectal vascularisation after mechanical anastomosis (Seracchioli et al., 2018). ICG identifies the vascularisation of a specific anatomical structure or tissue, showing vascular anatomy and local perfusion (Alander et al., 2012). After direct intrave-nous administration (0.25mg/Kg), a fast and objective evaluation of neoanastomosis vascularisation could be performed. When vascularisation is normal, the ICG turns fluorescent (dark green) once excited with a light in the NIR spectrum (De Neef et al., 2018). Although some prospective studies show less leakage incidence when this technique is applied (compared to overall rate) (Jafari et al., 2015; Kawada et al., 2014; Blanco-Colino and Espin- Basany, 2018), evidence quality is still poor and scant to recommend their use routinely in bowel DIE surgical cases and must remain as a part of analytical, experimental protocols to demonstrate their real benefit. Currently, there is an ongoing clinical trial conducted by the group of Clermont Ferrand assessing the potential role of ICG in reducing fistula rates after rectal shaving surgery.

## c. Surgical technique

### General and specific surgical principes for Bowel anastomosis

General surgical principles and technical points for bowel closure must be followed and maintained to avoid anastomotic leakage (Nezhat et al., 2018; De Cicco et al., 2011) (Table V).

**Table V:** Surgical principles and technical points for bowel anastomosis construction.

Area	Factors	Rationality
General Principles	Adequate tissue perfusion	Correct perfusion of anastomotic line is necessary for correct wound healing and prevention of micro and macro leakages
	Tension free	Since tension reduce the blood flow at the suture line, adequate bowel mobilization is required for leave the anastomotic line free of tension
	No tissue overlapping	Tissue overlap increase the risk of fistulas and must be avoided in single-layer closures. Flat knots are necessary to avoid this problem
	Minimize tissue trauma	Minimum trauma reduce the risk of microbial colonization, keep an adequate blood supply and faster the wound healing
	Adequate Hemostasis	Precise and complete hemostasis prevents post-operative hematomas and/or seromas which can interfere with the correct tissue apposition necessary for complete union of wound edges
	No wound dead spaces	Dead space are responsible of inadequate wound tissue approximation and accumulation of serum or blood, impairing wound healing and predisposing to infection
	Removal of foreign and necrotic tissue	Direct and complete apposition of wound edges is necessary and must be free of any other tissues or foreign body
Bowel Closure	Monofilament threads	Single strand sutures are resistant to harboring organism, reducing the capillarity effect and therefore the risk of infection. In case of entering the rectum, risk of bacterial proliferation is reduced
	Round needles	It penetrates the tissue by spreading without cutting it. It is the recommended for gastrointestinal surgery due their specific sharpness and smoothly tissue penetration, preventing leakage
	Specific surgical technique	Analysis of surgical factors will be discussed later

### Stapler versus hand-made anastomosis

Excluding ileocolic and oesophago-gastric anastomosis evaluations, three significant meta- analyses have been published in recent years. Naumann et al. (2015) evaluated the risk of leak, abscess, and fistula after bowel anastomosis in the emergency setting. In seven studies (5 retrospectives, one RCT, and one prospective non-randomised) and more than 1,205 anastomoses, there were no significant differences between the handsewn and the stapler technique in the risk of AL (OR:1.00). Furthermore, Neutzling et al. (2012) presented their results specifically for colorectal anastomosis, including 9 RCTs and 1233 patients. No significant differences were seen in both clinical (6.3% vs. 71%) and radiological (7.8% vs. 7.2%) anastomotic dehiscence between the arms, regardless of the level of the anastomosis. Finally, a systematic review of eleven systematic reviews concerning handsewn versus stapled anastomosis reported no evidence of the superiority of any specific technique (Hemming et al., 2013). The conclusion is that the decision of the type of anastomosis is likely a matter of surgeon’s preference and experience, as techniques appear to be substantially equivalent concerning leak rate. However, in the case of stapler use, recent retrospective evidence supports the fact that the number of cartridges fired is a relevant factor for AL occurrence, significantly increasing when three or more cartridges are used (Braunschmid et al., 2017).

### Anastomosis reinforcement

#### 1. Bio-absorbable staple-line

The use of bio-absorbable staple-line reinforcing material is appealing to some. Although studies have shown that these reinforcements are safe and there have been several RCTs on the subject, to date, there have been no compelling studies which have demonstrated a decrease in leakage rates when they are used (Placer et al., 2014).

#### 2. Sutures

**Figure 5:**
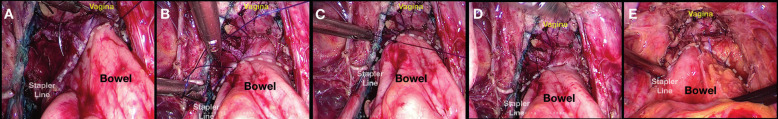
Suture reinforcement of anastomosis. 5 consecutive images showing the manual suture reinforcement after stapler anastomosis. Using delayed absorbable or nonabsorbable mono-filament sutures, 1 to 5 intra-corporeal stitches are performed to secure the anastomosis strength against AL during the first days PO. A,B,C and D: Reinforcement stitch with a triple-double blocking sequence; E: Final view after 5 stitches.

Reinforcement sutures are typically placed around the anastomosis, but intra-luminal reinforcement has also been carried out (Kim et al., 2015). To date, there is no compelling evidence indicating that suture rein-forcement reduces leakage, yet these techniques may improve a surgeon’s confidence regarding the strength of one’s anastomosis (Figure 5).

#### 3. Fibrin glues

To date, one RCT has failed to show a decrease in leakage rate with the use of fibrin glue (Silecchia et al., 2008). At the same time, there have been several case series showing low meagre anastomotic leakage rates with the use of these glues (Lee et al., 2004). Fibrin glue application over the stapled anastomosis was found not to be significantly associated with leakage following laparoscopic rectal cancer surgery without stool diversion (Huh et al., 2010).

### Suture material

Decades ago, several materials, such as silk, linen, catgut, polyglactin 910, and nylon, were commonly used for colorectal anastomosis. Nowadays, it is evident that absorbable sutures are safe, leaving no channel for luminal microbial migration once absorbed. Most gastrointestinal anastomoses, including colorectal, are constructed with polydioxanone sutures.

Absorbable sutures compared with non- absorbable or slowly absorbable sutures cause more tissue reaction and dissolve too rapidly, reducing the anastomotic strength (Van Winkle et al., 1975).

Multifilament, compared to monofilament sutures, cause more tissue damage and easier adherence of material within the interstices of the multifilament (Deveney and Way 1977), creating a risk of infection (Durdey and Bucknall 1984).

Polydioxanone thread possesses all characteristics considered important; monofilament, little tissue reaction, slowly absorbable with long preservation of strength and low bacterial adherence risk. Based on experimental studies, non-absorbable, or slowly absorbable monofilament sutures seem to be the first choice for colorectal anastomosis (Slieker et al., 2013).

### Suture format

#### 1. Interrupted vs continuous fashion

Continuous suture provides a tighter seal than an interrupted one. The main fear is that if this suture breaks, the entire suture line could open. RCTs investigating interrupted and continuous sutures for colorectal anastomosis are lacking. Only one small, non-randomised, comparative clinical study found no significant differences (Houdart, 1994). Two clinical and experimental studies have not concluded that one technique is superior to the other and a high level of evidence is lacking; however, from a technical and time-consuming point of view, a continuous suture is preferable over interrupted sutures (Slieker et al., 2013).

#### 2. Size of suture bites

Lembert described the construction of intestinal anastomoses in dogs using suture bites with 5-mm distance to the cut edge nearly two centuries ago (Breschet, 1828). An RCT allocated patients to have bowel sutures placed either 5 or 10 mm from the cut edges, with no significant differences in leakage rates (Greenall et al., 1979).

#### 3. In-between distance of bites

Lembert reported a distance of approximately 1 cm between sutures (Breschet, 1828). Neither comparative clinical studies nor cohort studies were found. Animal experiments indicate that small distance between sutures (1.5 mm) improves apposition compared with a more considerable distance (2.5 mm) (Waninger et al., 1992). However, due to lack of clinical studies on this topic, there is no precise conclusion in the literature regarding this issue.

#### 4. Suture tension

In routine clinical practice, two undefined schools of thought seem to exist. The first believes that sutures should be tightened to prevent dehiscence of the anastomosis, and the second considers that sutures should be applied more loosely, allowing maximal perfusion of the cut edges. The bowel is highly supplied with blood and may become oedematous and hardened when tight stitches are used. Only one rat study investigated this, with moderate tension giving the best histological and micro-angiographic results (Waninger et al., 1992). Whether pressure on knots could influence the incidence of AL in a clinical setting has not been investigated, and therefore, the optimal tension on the thread or the knot is unknown.

#### 5. Thickness of the bite

The main strength of the gastrointestinal tract is in the muscularis and submucosa. Thus, effective closure involving at least these two layers is needed. Additionally, avoiding entering into the mucosa could help to prevent leakage. Cohort studies report low rates of AL for both serosa-submucosal and full-thickness suture types (Leslie and Steele 2003). We can conclude that both serosa-submucosal and full-thickness sutures seem to provide low rates of leakage.

#### 6. Inverting vs everting

Both everting and inverting (as well as end-to-end) techniques have been performed, but both have drawbacks. An RCT showed a 5-fold increased incidence of AL in patients receiving an everting compared with those receiving an inverting suture. Hence, there seems to be an advantage of inverting over everting colorectal anastomosis (Goligher et al., 1970).

#### 7. Single vs double-layer anastomosis

The classic technique is based on a double-layer inverting anastomotic method. One RCT (Everett, 1975) matched the inclusion criteria, showing no significant differences in AL between single- and double-layer colorectal anastomosis in 92 patients. However, in the subgroup analysis of low colorectal anastomosis, the incidence of AL in those with the double-layer technique was significantly higher. Single-layer anastomosis has the additional advantage of being less time consuming to perform and is less costly (Burch et al., 2000); hence the published literature favourings single layer anastomosis.

### Use of drainage

#### a. Pelvic drainages

A pelvic drain may prevent haematomas or seromas that constitute a medium for bacterial infection which can involve the anastomosis, thereby causing dehiscence. Moreover, a pelvic drain may help control leaks if they do occur, leading to a less severe clinical course (Qu et al., 2015). However, routine prophylactic use is debatable (Emile and Abd El-Hamed, 2017). Two retrospective studies found pelvic drainage associated with low-er rates of AL, though without reaching statistical significance (Akiyoshi et al., 2011). However, the lack of pelvic drain was found to be independently predictive of leakage at multivariate analysis. Two main systematic reviews of RCTs have been published. A Cochrane meta-analysis included 6 RCTs and over 1100 patients, and reported no reduction of leakage after prophylactic use of drainage (Jesus et al., 2004). A recent meta-analysis including RCTs and retrospective studies indicate a significant decrease (49%) in AL when drains are used in rectal infra- peritoneal bowel resections. However, when RCTs were analysed alone, this reduction did not persist (Rondelli et al., 2014).

#### b. Trans-anal drainages

The evidence is contradictory. One RCT showed no reduction in AL when trans-anal stents were used in 194 patients subjected to anterior rectal resection (Bulow et al., 2006). Nevertheless, recent prospective and retrospective studies show that the use of a trans- anal drainage tube significantly reduces AL and other unfavourable effects of post-operative diarrhoea (Tanaka et al., 2017; Nishigori et al., 2014; Zhao et al., 2013) by lowering endo-luminal pressure (gas and fluids) at the anastomotic line in the early period. Despite this, their systematic use remains questionable and is a matter of preference of the surgeon.

### Omentoplasty

Theoretically, the interposition of omental graft on a vascular pedicle, covering the area of the anastomosis, offers two main benefits (Wiggins et al., 2015; Hayari et al., 2004):

1. *Re-enforcement of the anastomotic line* during the first post-operative days (when there is a higher risk of leakage,) acting as a biologically viable plug which can seal microscopic leaks.
2. *Increased angiogenesis and neo-vascularisation at the anastomotic site* by providing vascular endo- thelial growth factor, promoting microvascular anastomosis between the omentum and the bowel wall (Adams et al., 1992).

**Figure 6:**
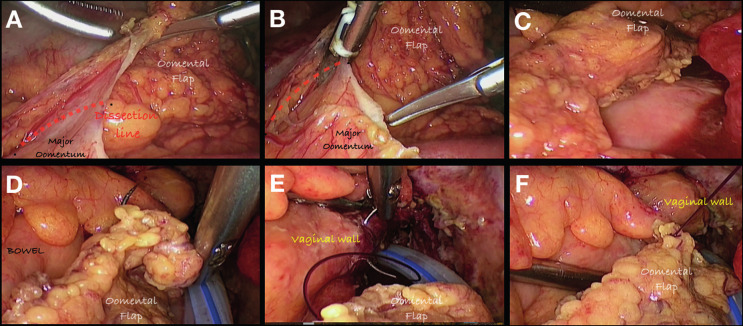
Omentoplasty. Sequence of 6 images showing the dissection of the major oomentum in order to create the oomental flap. A and B: Dissection line in order to create the flap ; C: Omental flap is done and ready to interpose. D and E: Fixation of the flap into the vaginal wall. F: Final position of the flap between the bowel and vaginal suture lines.

Limitation of their use is mainly due to the fear of omental necrosis and increased risk of recurrence in the cancer setting (van Garderen et al., 1991) (Figure 6).

In the prospective series of Ozben et al. (2016), no reduction in AL or surgical site infection rates was seen when the omental flap was used after rectal cancer surgery. Moreover, the systematic review published by Wiggins et al. (2015) including three RCTs and 943 colorectal anastomoses showed no significant differences in the rate of leakage (5% vs 8.4%), in-hospital mortality (4.2% vs 4.1%) and anastomotic stricture (1.9% vs 5%) between patients with and without omentoplasty.

## Conclusions

Anastomosis leakage is the leak of intra-luminal content from the suture line between 2 hollow viscera. It is a heterogeneous pathology by definition, but severe in its nature, causing severe morbidity, re-admissions, re-operations, a more extended hospital stay, lower quality of life and up to 15% mortality.

All types of bowel endometriosis surgical treatment carry a risk of leakage and RVF, and even when these complications are predominantly seen in the segmental resection group, with an overall risk of 1.7% (0-6%) and 3.9% (0-10.3%), evidence is predominantly retrospective and AL definitions are heterogeneous among the different studies. Careful monitoring is essential after any of these procedures. Control of modifiable risk factors, together with strictly following surgical principles such as avoidance of anastomotic tension, tissue ischemia and overlapping remain paramount in general prevention.

Results of this evidence-based analysis lead us to recommend the following peri-operative modifiable measures; the use of either stapler or handsewn (single layer closure) anastomosis construction; intra-operative use of air leak test to check the mechanical integrity of anastomotic line; systematic use of pelvic (in infra-peritoneal anastomosis) and trans-anal drainage; application of further preventive interventions (protective or ghost ileostomy) when the nodule is located under 8 cm from the anal verge and in high-risk patients; closure of the vagina before performing bowel resection (when colpotomy is required); systematic use of non-absorbable oral antibiotics one day before surgery and performing partial mesorectal resection near the bowel wall (Table VI).

**Table VI:** Summary of recommendations for main risk factors and preventive techniques of anastomotic leakage.

Procedure	Rationality	Evidence - CTF	Recommendation
NSAIDs Use	Down regulation of prostaglandins expression and corresponding hydroxypro- line levels, harming the healing process	I	Significant increase of leakage. Use with caution in patients with predisposeding factors of anastomotic leakage
Bowel preparation (mechanical)	Reduce material load and intestinal microbiome related to anastomotic leakage	I	Avoid mechanical preparation since it does not reduce the risk of leakage, increase electrolytic disturbances and infections
Bowel preparation (oral)	Reduce material load and intestinal microbiome related to anastomotic leakage	I	Use non-absorbable oral antibiotics one day before surgery
Tumor size	Bigger tumors determine longer resections enhancing the risk of anastomotic complications	II.1	Studies focussed in oncologic setting. Nodules over 3 cm more often require segmental resection, increasing the leakage risk
Level of anastomosis	Lower rectal anastomosis is in higher risk of leak due the lack of serosal layer	II.1	Consider further preventive interventions (protective or ghost ileostomy, omentoplasty, others) when positioned under 8 cm to the anal verge
Stapler or handsewn	Type of anastomosis could predisposed to leakage	I	Select according to surgeon preference and experience. No differences in leak rates. Shorter operative times in stapler tenhnique
Numbers of layers (closure)	Number of layers can modify the risk of leakage by determining mechanical strength, tissue ischemia and overlapping	I	Single layer closure significantly reduce risk of leakage in low colorectal anastomosis, as well as operative time and costs
Type of threads	Use of delayed-absorbable or non-absorbable monofilament threads reduce tissue reaction, damage and adherence of materials	II.2	Prefer polydiaxonone threads. Avoid rapid/ normal absorbable threads
Bowel closure fashion	Specific suture technique may reduce the risk of leakage	II.2	No differences in risk of leakage. Prefer continuous inverting sero-submucosal or full-thickness stitches
Anastomosis reinforcement	Intra or extraluminal suture reinforcement could enhance anastomotic line strength	II.1	Benefits have not been demonstrated either for sutures, fibrin glues or bio-absorbable stapler. Use prudently
Mesorectal resection	Total mesorectal resection impair local bowel irrigation predisposing anastomotic line necrosis and leak	I	Perform partial mesorectal resection as near as possible to bowel and no more than 2 cm from endometriosis nodule. If TME is done, consider additional leakage protective techniques
Concomitant vaginal resection	Anatomical predisponding factor for RVF	II.1	Always close the vagina before performing bowel resection. Interposition of omental flap is recommended
Air leak test	Direct evaluation of mechanical anastomosis competence and micro-leaks could reduce AL	I	Systematic use is recommended since further procedures in a positive test reduce significantly the leak
Omentoplasty	Increase angiogenesis and neovascularization - Act as a biologically viable plug that can seal microscopic leaks.	I	Does not significantly reduce lekage. Minor risk of flap necrosis.Use prudently.
Pelvic drainage	Prevent haematomas or seromas which could predispose to infection and cause anastomotic dehiscence	I	Significant leakage reduction in rectal infra peritoneal anastomosis. Prefer to use in those cases. No differences in other levels
Transanal drainage	Prevent haematomas or seromas which could predispose to infection and cause anastomotic dehiscence	I	Use following surgeons experience and criteria. Evidence favor their use since reduce leakage and diarrhoea rates.

Temporary defunctioning stomas may decrease the morbidity and clinical consequences of the leakage in over 65% of low colorectal anastomosis, but are associated with significant side effects that must be balanced against the risk of leakage. The treatment, considering the benign nature of endometriosis, must always be tailored according to the patient’s disease, desires and expectations, with comprehensive case-by-case selection and patient counselling.

Finally, readers must be aware that the majority of the studies on this topic come from colorectal surgeons’ experience. This is relevant since colorectal oncology patients usually have a different demographic to the young, healthy patients in the endometriosis setting. However, the large endometriosis series, including more than 5500 segmental resections, support the conclusions presented here.
